# Differential role of melatonin in healthy brain aging: a systematic review and meta-analysis of the SAMP8 model

**DOI:** 10.18632/aging.202894

**Published:** 2021-04-02

**Authors:** Dewan Md. Sumsuzzman, Zeeshan Ahmad Khan, Jeonghyun Choi, Yonggeun Hong

**Affiliations:** 1 Department of Rehabilitation Science, Graduate School of Inje University, Gimhae 50834, Korea; 2 Biohealth Products Research Center (BPRC), Inje University, Gimhae 50834, Korea; 3 Ubiquitous Healthcare and Anti-Aging Research Center (u-HARC), Inje University, Gimhae 50834, Korea; 4 Department of Physical Therapy, College of Healthcare Medical Science and Engineering, Gimhae 50834, Korea; 5 Department of Medicine, Division of Hematology/Oncology, Harvard Medical School-Beth Israel Deaconess Medical Center, Boston, MA 02215, USA

**Keywords:** melatonin, brain aging, oxidative stress, senescence, meta-analysis

## Abstract

The relationship between oxidative stress (OS) and cellular senescence (CS) is an important research topic because of the rapidly aging global population. Melatonin (MT) is associated with aging and plays a pivotal role in redox homeostasis, but its role in maintaining physiological stability in the brain (especially in OS-induced senescence) remains elusive. Here, we systematically reviewed the differential role of MT on OS-induced senescence in the SAMP8 mouse model. Major electronic databases were searched for relevant studies. Pooled mean differences (MDs)/standardized mean differences (SMDs) with 95% confidence intervals (CIs) were calculated to estimate the effect size. Overall, 10 studies met the inclusion criteria. MT treatment was associated with the reduction of lipid peroxidation (SMD = −2.00, 95% CI [−2.91, −1.10]; *p* < 0.0001) and carbonylated protein (MD = −5.74, 95% CI [−11.03, –0.44]; *p* = 0.03), and with enhancement of the reduced-glutathione/oxidized-glutathione ratio (MD = 1.12, 95% CI [0.77, 1.47]; *p* < 0.00001). No differences were found in catalase and superoxide dismutase activities between MT-treated and vehicle-treated groups. Furthermore, nuclear-factor-κB, cyclin-dependent kinase-5, and p53 were regulated by MT administration. MT may improve physiological stability during aging by regulating interactions in brain senescence, but acts differentially on the antioxidant system.

## INTRODUCTION

**Figure 1:**
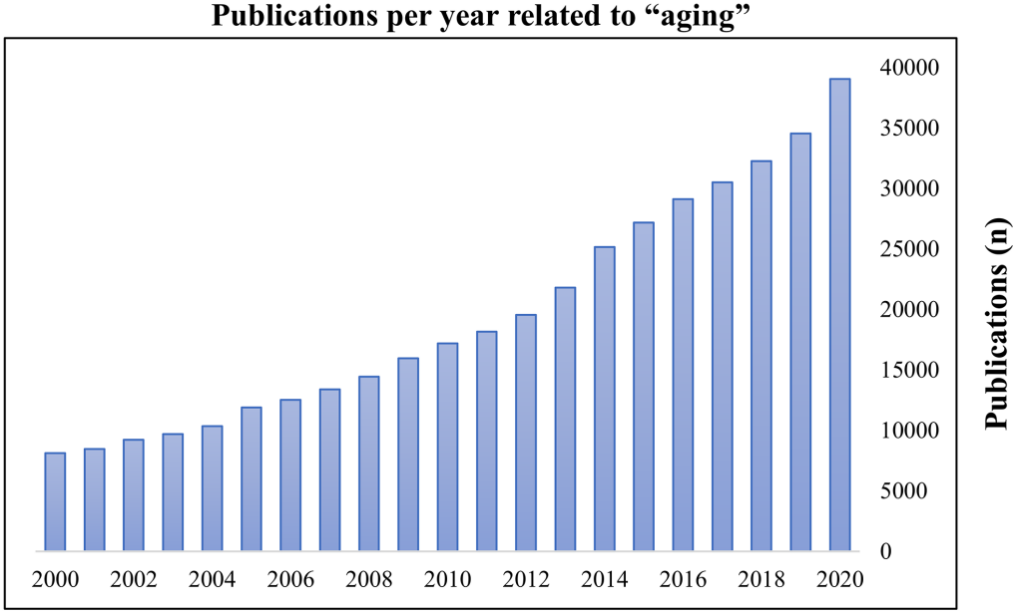
**Publications per year related to ‘aging’.** The search term ‘aging’ was entered on PubMed on 16 November 2020. Results were plotted as publications per year.

Aging is the greatest risk factor for nearly all diseases and is an important global healthcare challenge as the older adult group (age ≥ 60 years) is growing faster than younger age groups [1, 2]. Because of the considerable impact of aging on the overall population, research on healthy aging has become a common focus worldwide. Consequently, the publication frequency regarding aging has steadily increased in recent years (Figure 1). Recently, several hallmarks of aging have been identified. Of these, cellular senescence (CS) is an important topic in scientific research regarding aging processes [3, 4]. Disruption of redox homeostasis due to prolonged exposure to oxidative stress (OS), manifested as reactive oxygen species (ROS)-mediated damage to biomolecules, has been identified as a crucial mediator for the progression of CS [5–9]. Although selective elimination of senescent cells (via senolytics) and prevention/slowed progression of senescence features (via senostatics) are attractive strategies, they also have undesirable adverse-effects such as high toxicity, low bioavailability, and chemical instability [10–12]. Therefore, the roles of biomolecules in maintenance of physiological homeostasis between OS and CS may provide novel strategies to promote healthy aging.

During the aging process, the brain is presumably the organ most prone to free radical damage because of its high oxygen utilization, high concentrations of polyunsaturated fatty acids, and low concentrations of cytosolic antioxidants [13]. Lipid peroxidation (LPO) is oxidative damage to lipids induced by ROS, whose reaction with polyunsaturated fatty acids in cell membranes has been proposed as a critical mechanism involved in cellular aging [14]. Moreover, protein carbonylation is an irreversible oxidative protein modification process in cells, organelles, and tissues, which increases with age [15]. Its aggregation can become toxic to living cells and has been directly linked to a large number of age-related disorders [15, 16]. Besides, cathepsin-B (lysosomal thiol proteases) expression increases proportionally with age and its subcellular distribution is apparently altered, thus resulting in elevated production of mitochondria-mediated ROS [17]. Furthermore, the activation of several signaling pathways, including those of nuclear factor-κB (NF-κB), cyclin-dependent kinase 5 (Cdk5), and p53, play pivotal roles in the promotion of OS-induced CS [18–20]. Taken together, these findings have significant implications for the understanding of how age-related induction of OS is directly linked with premature senescence (i.e., stress-induced premature senescence).

The senescence-accelerated mouse prone 8 (SAMP8) strain is an excellent mammalian model to study OS-induced senescence-related impairments and degeneration in the brain [21–24]. The overexpression of alpha-synuclein together with phosphorylated tau protein significantly reduces antioxidant machinery in SAMP8 [25–27], indicating that OS may contribute to senescence-dependent brain impairments. Importantly, the SAMP8 mouse model expresses premature senescence and has features similar to those of aged humans, such as shortened lifespan and diminished physical activity [28]. The SAMP8 also exhibits age-related deterioration of learning and memory [29, 30], as well as key pathological features that induce premature senescence, including OS [29, 31]. The SAMP8 mouse is a natural aging animal model, rather than a transgenic model of aging or age-related diseases. This suggests that underlying mechanisms must be linked to premature senescence and more closely represent the complex multifactorial nature of aging. Furthermore, the lifespan of SAMP8 mice (10–17 months) is significantly shorter than that of normal laboratory mice (22–36 months) [32, 33]. Therefore, SAMP8 mice provide the best alternative aging research model in terms of time and cost.

Melatonin (MT), an endocrine hormone of the pineal gland, can directly scavenge free radicals and was previously identified as a potent antioxidant [34–37]. MT also stimulates antioxidative enzymes such as superoxide dismutase (SOD) and glutathione peroxidase (GPx); this action further diminishes the cellular oxidation state [34, 38]. A previous study demonstrated that oral administration of MT to SAMP mice was protective against age-related oxidative DNA damage in the brain [39]. Another study revealed that MT treatment inhibited age-related increases in both LPO and protein oxidation in SAMP8 mice [40]. Although many studies have demonstrated the protective effects of MT in several aging animal models, the mechanism underlying the effects of MT in SAMP8 mice remains unclear. Furthermore, a systematic review of the literature and meta-analysis of preclinical data can demonstrate the quality and strength of existing research. However, no such study has yet been conducted to evaluate the efficacy of MT in the SAMP8 mouse model. Thus, this systematic review assessed previous research concerning the protective effect of MT against brain aging in the SAMP8 mouse model. This analysis is intended to provide evidence to support the therapeutic potential of MT to promote healthy aging and prevent age-related diseases by maintenance of physiological homeostasis between OS and CS.

## RESULTS

### Study search and selection

**Figure 2:**
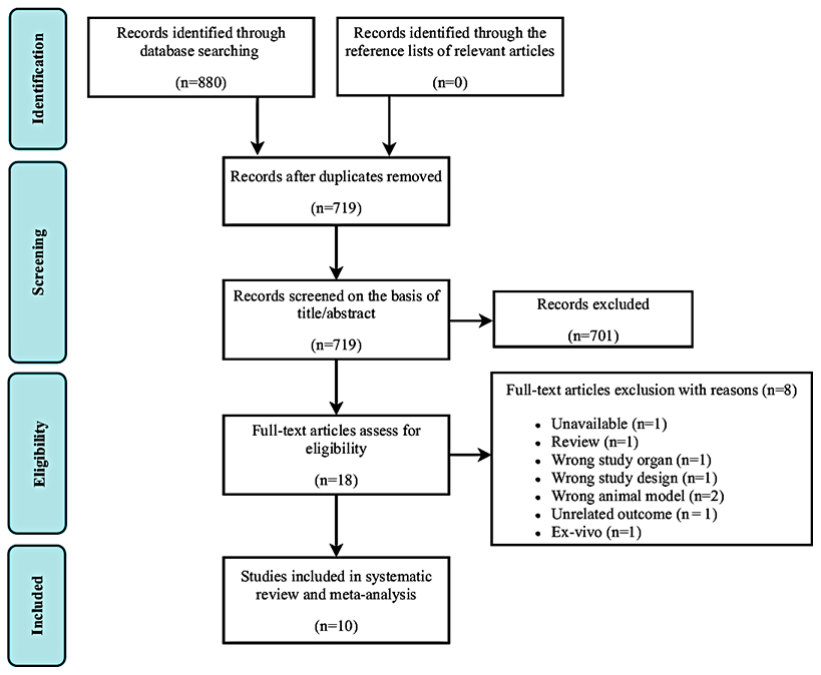
**Flow diagram of study search and retrieval process.**

In total, 880 studies were found through an electronic database search. After removal of duplicate studies, the titles and abstracts of 719 potentially relevant articles were screened. Of these, 701 were excluded based on title and abstract screening. The remaining 18 studies were included in the full-text screening. Of these, eight studies were excluded for the following reasons: unavailable data (n = 1), review article format (n = 1), inappropriate study organ (n =1), inappropriate study design (n = 1), inappropriate animal model (n = 2), unrelated outcome (n = 1), and *ex vivo* design (n = 1). Finally, 10 studies [40–49] fulfilled the eligibility criteria and were selected for systematic review and meta-analysis (Figure 2).

### Study characteristics

The main characteristics of the studies that assessed the effect of MT in the SAMP8 model are reported in Table 1. These studies were published between 2002 and 2011. In nine of the 10 included studies, MT was administered in the drinking water at a dose of 10 mg/kg/day. In the remaining study, MT was administered at a dose of 1 mg/kg/day subcutaneously [45]. Eight studies administered MT when animals were 2–10 months of age [41–44, 46–49], one study administered MT when animals were 7–12 months of age [40], and the remaining one study divided into mice into two MT treatment groups based on age (group 1: 4–8 months; group 2: 7–11 months) [45]. Five studies included both male and female mice [40–42, 48, 49], and three of these reported sex-specific results [41, 42, 48]. Three studies used only male mice [43, 45, 46] and the remaining two studies did not report animal sex [44, 47].

**Table 1:** Characteristics of the included studies.

**Author (Year)**	**Age**	**Sex**	**Con group** **(n)**	**MT group** **(n)**	**ROA**	**Dose** **(MT)**	**Duration**	**Evaluated parameters**
Caballero B et al. [47] (2009)	Offspring of SAMP8	NR	4	4	Oral (drinking water)	10 mg/kg	From 2 months to 10 months of age	Brain oxidative stress markers: (SOD, GRx, CAT in cerebral tissue); lysosomal proteases activities: (cathepsin B, cathepsin D); molecular factors: (Bcl-2, p53 by western blot)
Caballero B et al. [44] (2008)	One month of SAMP8	NR	4	4	Oral (drinking water)	10 mg/kg	From 2 to 10 months of age	Brain oxidative stress markers: (carbonylated Protein in the brain); molecular factors: (NF-kB in the whole brain by western blotting; immunohistochemistry for NF-kB)
Carretero M et al. [48] (2009)	Offspring of SAMP8	Both	Male=9 Female=9	Male=9 Female=8	Oral (drinking water)	10 mg/kg	From 2 months to 10 months of age	Brain oxidative stress markers: (LPO, nitrite, GPx, GRx, GSH/GSSG ratio)
Cheng S et al. [45] (2008)	Offspring of SAMP8	Male	10	A. MT-4^†^ group=10 B. MT-7^††^ group=10	Subcutaneous	1 mg/kg/day	A. MT-4 group: from 4 to 8 months of age B. MT-7 group: from 7 to 11 months of age	Morphological factors: cresyl violet staining in the hippocampal CA1 and CA3 regions
García JJ et al. [49] (2011)	Offspring of SAMP8	Both	14	16	Oral (drinking water)	10 mg/kg	From 2 months to 10 months of age	Brain oxidative stress markers: (LPO, carbonylated Protein, GSH/GSSG ratio); lysosomal proteases activities: (cathepsin B, cathepsin D)
*Gutierrez-Cuesta J et al. [46] (2008)	Offspring of SAMP8	Male	16	16	Oral (drinking water)	10 mg/kg	From 2 months to 10 months of age	Molecular factors: (SIRT1 acetylated p53, acetylated NFκB in the brain by western blot
Gutierrez-Cuesta J et al. [43] (2007)	One month of SAMP8	Male	16	16	Oral (drinking water)	10 mg/kg	From 2 to 10 months of age	Brain oxidative stress markers: (LPO, carbonylated Protein, CAT in the cerebral cortex); morphological factors: Nissl stain; molecular factors: (immunohistochemistry for cdk5; cdk5, p35, GSK3, tau phosphorylation in the cortex by western blotting)
Nogués MR et al. [41] (2006)	One month of SAMP8	Both	Male= 8 Female=8	Male= 8 Female=8	Oral (drinking water)	10 mg/kg	From 2 to 10 months of age	Plasma oxidative stress markers: (LPO, GRx)
Okatani Y et al. [40] (2002)	Offspring of SAMP8	Both	16	15	Oral (drinking water)	10 mg/kg	From 7 to 12 months of age	Brain oxidative stress markers: (LPO, carbonylated Protein, SOD, GPx in cerebral tissue)
Rodríguez MI et al. [42] (2007)	Offspring of SAMP8	Both	Male=6 Female=6	Male=6 Female=6	Oral (drinking water)	10 mg/kg	From 2 to 10 months of age	Plasma inflammatory markers: (IL-1β, IL-2, IL-4, IL-5, IL-10, IFN-γ and TNF-α)

^†^melatonin treatment started from 4 months of age.

^††^melatonin treatment started from 7 months of age.

^*^the sample size was obtained from the corresponding author.

Con, control group; MT, melatonin group; n, sample size; ROA, route of administration; SAMP8, senescence-accelerated mouse prone 8; NR, not reported; SOD, superoxide dismutase; GRx, glutathione reductase; CAT, catalase; Bcl-2, B-cell lymphoma 2; NF-kB, Nuclear factor-κB; LPO, lipid peroxidation; GPx, glutathione peroxidase; GSH/GSSG, reduced glutathione/oxidized-glutathione ratio; SIRT1, sirtuin 1; cdk5, cyclin Dependent Kinase 5; GSK3, glycogen synthase kinase 3; IL-1β, interleukin-1β; IL-2, interleukin-2; IL-4, interleukin-4; IL-5, interleukin-5; IL-10, interleukin-10; IFN-γ, interferon-gamma; TNF-α, tumor necrosis factor-α.

### Risk of bias and quality of reporting

**Figure 3:**
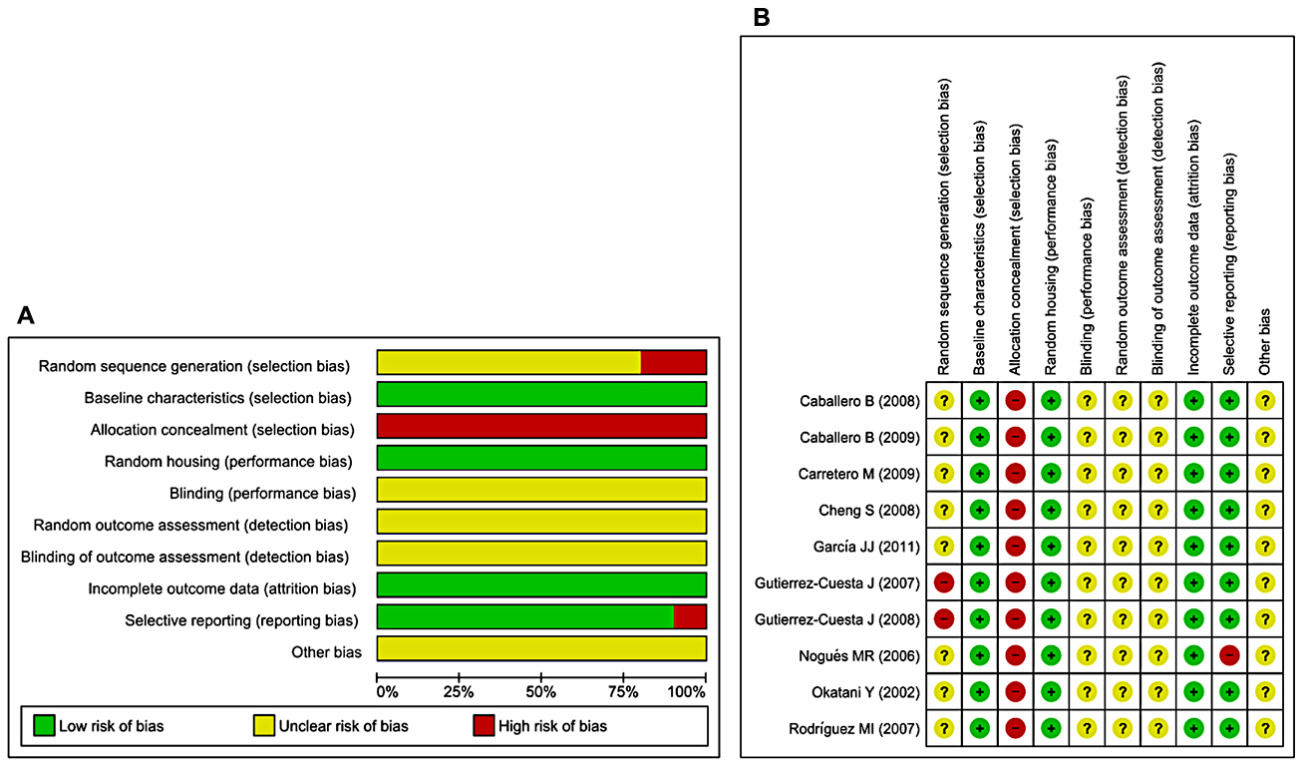
**Risk of bias.** (**A**) Overall RoB for each item in the SYRCLE tool for all included studies. Each RoB item is presented as a percentage based on all included studies. (**B**) Individual RoB for each of the included animal studies. Each item in the SYRCLE tool was scored as ‘yes’, ‘no’, or ‘unclear’.

The abridged risk of bias (RoB) assessment used in this study is presented in Figure 3A, and the individual RoB scores for each study are presented in Figure 3B. Randomization is considered a fundamental measure to reduce bias, but is rarely reported in preclinical trials. Eighty percent of the included studies reported random allocation of the animals, although no study sufficiently specified the method of randomization. Baseline characteristics and random housing were often considered indicative of low RoB. However, blinding of the investigators and caregivers, random outcome assessment, and blinding of outcome assessment items were frequently rated as unclear RoB. The rating ‘unclear’ was defined as insufficient reporting of most of the relevant criteria that are considered essential for the assessment of preclinical trials. Selective outcome reporting and incomplete outcome data were considered indicative of low RoB (90–100%). Allocation concealment was considered indicative of high RoB. Three studies were presumed to have high RoB because of random sequence generation/selective outcome reporting [41, 43, 46]. During extraction of raw data from included studies, we most often used GetData Graph Digitizer, thereby other bias parameter was rated as unclear RoB. Inadequate reporting of the measures used to reduce bias was reflected in our RoB assessment because numerous items were scored as unclear.

### Meta-analysis

### *Effects of MT on LPO and carbonylated protein*

**Figure 4:**
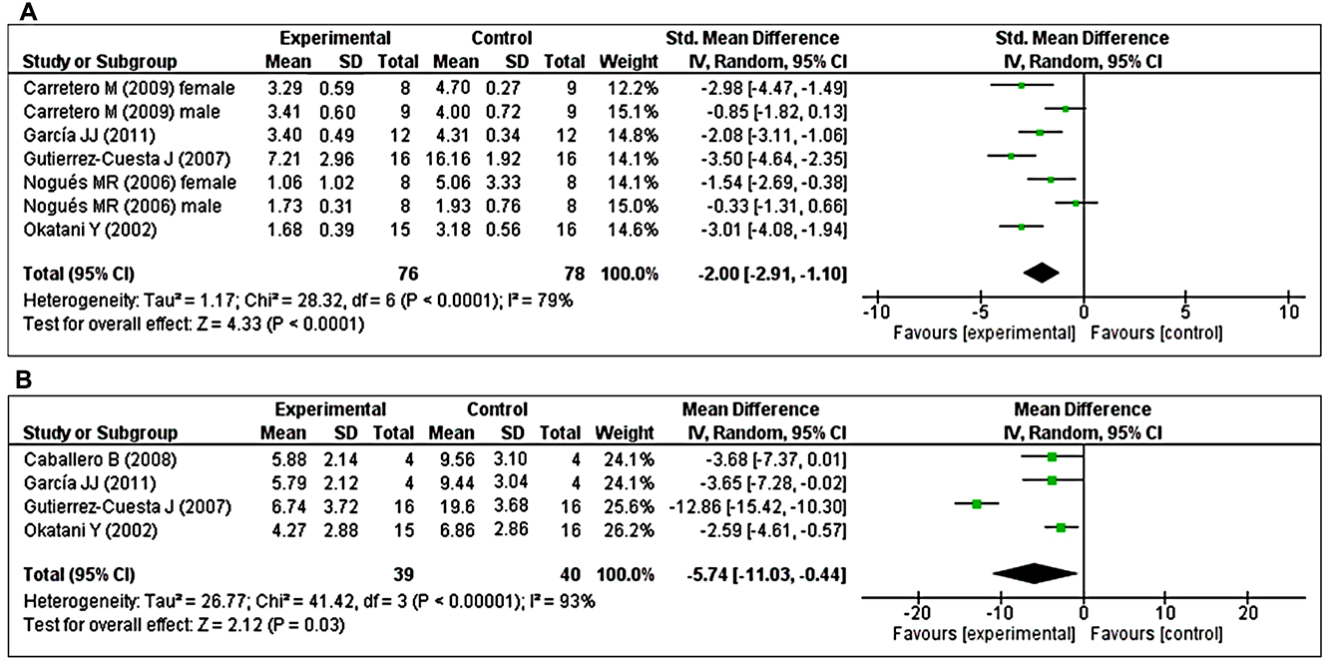
**Forest plot comparing changes in the abundance of LPO and carbonylated protein levels between MT-treated and vehicle-treated groups.** Compared with vehicle treatment, (**A**) LPO and (**B**) carbonylated protein levels were both significantly reduced in the MT-treated group. The unit for LPO almost in all studies is nmol/mg, except Nogues MR et al*.* (nmol/ml). For carbonylated protein the unit is nmol/mg. The prism represents the overall statistical results of the experimental data, squares represent the weight of each study, and horizontal lines represent the 95% CIs for each study. MT, melatonin; LPO, lipid peroxidation; CI, confidence interval; SD, standard deviation; IV, independent variable.

The effects of MT on LPO were determined in five studies [40, 41, 43, 48, 49]. Of these, two studies [41, 48] investigated the effects of prolonged MT administration on changes in the abundance of LPO, separately, in male and female groups. Using a random-effects model, we found that prolonged oral MT administration significantly reduced LPO levels (I^2^ = 79%; SMD = –2.00; 95% CI [–2.91, –1.10]; *p* < 0.0001) (Figure 4A). Furthermore, four studies [40, 43, 44, 49] investigated the effects of prolonged MT administration on changes in the abundance of carbonylated protein levels. Using a random-effects model, we found that prolonged oral MT administration significantly reduced carbonylated protein levels (I^2^ = 93%; MD = –5.74; 95% CI [–11.03, –0.44]; *p* = 0.03) (Figure 4B).

### *Effects of MT on changes in antioxidant enzyme activity*

**Figure 5:**
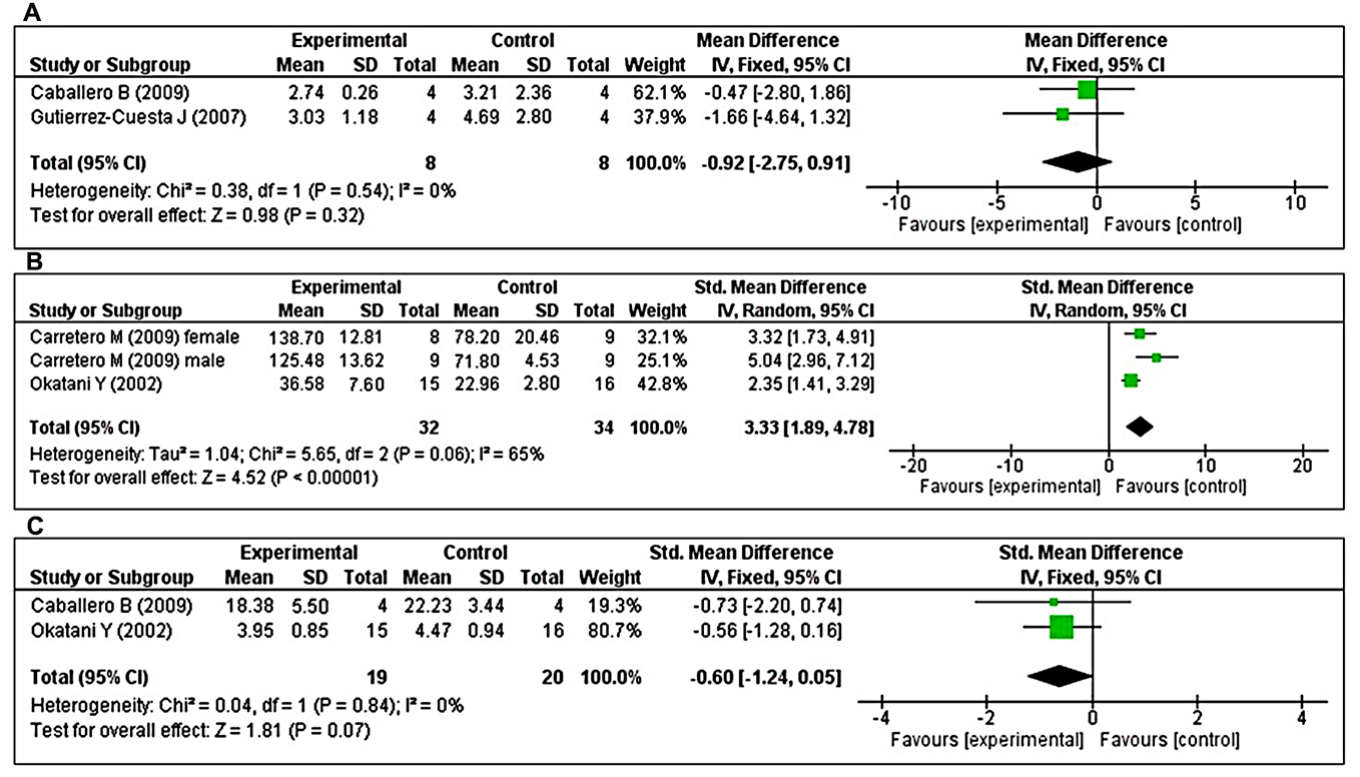
**Forest plot comparing changes in antioxidant enzyme activity between MT-treated and vehicle-treated groups.** Compared with vehicle treatment, (**A**) CAT activity was unchanged in the MT-treated group, (**B**) GPx activity was significantly enhanced in the MT-treated group, and (**C**) SOD activity did not differ in the MT-treated group. The unit for catalase and SOD are μmol/mg, and U/mg, respectively. For GPx, the unit is nmol/min/mg in Carretero M et al*.* and mU/min/mg in Okatani Y et al*.* The prism represents the overall statistical results of the experimental data, squares represent the weight of each study, and horizontal lines represent the 95% CIs for each study. MT, melatonin; CAT, catalase; GPx, glutathione peroxidase; SOD, superoxide dismutase; CI, confidence interval; SD, standard deviation; IV, independent variable.

The effects of MT on major antioxidant enzymes including catalase (CAT), GPx, and SOD were assessed in two studies each. Additionally, the glutathione reductase (GRx) and reduced glutathione/oxidized glutathione (GSH/GSSG) ratio were evaluated in three and two studies, respectively. Two studies [43, 47] investigated the effects of prolonged MT administration on changes in CAT activity. Using a fixed-effects model, we found that prolonged oral MT administration did not change CAT activity (I^2^ = 0%; MD = –0.92; 95% CI [–2.75, 0.91]; *p* = 0.32) (Figure 5A). Two studies [40, 48] investigated the effects of prolonged MT administration on changes in GPx activity. Of these, one study [48] investigated the effects of prolonged MT administration on changes in the abundance of GPx, separately, in male and female groups. Using a random-effects model, we found that prolonged oral MT administration significantly enhanced GPx activity (I^2^ = 65%; SMD = 3.33; 95% CI [1.89, 4.78]; *p* < 0.00001) (Figure 5B). Two studies [40, 47] investigated the effects of prolonged MT administration on changes in SOD activity. Using a fixed-effects model, we found that prolonged oral MT administration did not significantly change SOD activity (I^2^ = 0%; SMD = -0.60; 95% CI [–1.24, 0.05]; *p* < 0.07) (Figure 5C).

**Figure 6:**
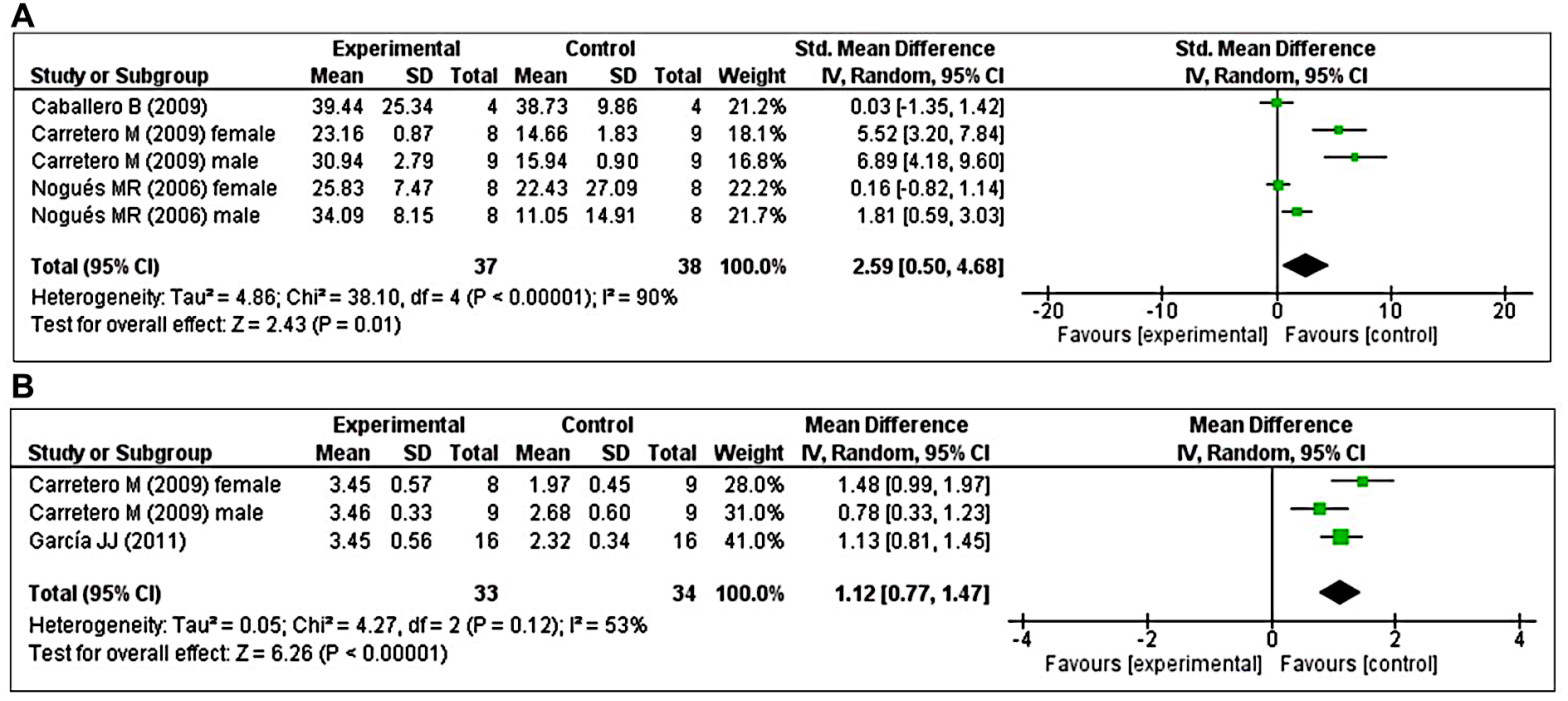
**Forest plot comparing changes in GRx activity and GSH/GSSG ratio between MT-treated and vehicle-treated groups.** Compared with vehicle treatment, (**A**) GRx activity and (**B**) GSH/GSSG ratio were both significantly enhanced in the MT-treated group. The unit for GRx almost in all studies is nmol/mg, except Nogues MR et al. (nmol/ml). The prism represents the overall statistical results of the experimental data, squares represent the weight of each study, and horizontal lines represent the 95% CIs for each study. MT, melatonin; GRx, glutathione reductase; GSH/GSSG, reduced-glutathione/oxidized-glutathione; CI, confidence interval; SD, standard deviation; IV, independent variable.

Three studies [41, 47, 48] investigated the effects of prolonged MT administration on changes in GRx activity. Of these, two studies [41, 48] investigated the effects of prolonged MT administration on changes in the abundance of GRx, separately, in male and female groups. Using a random-effects model, we found that prolonged oral MT administration significantly enhanced GRx activity (I^2^ = 90%; SMD = 2.59; 95% CI [0.50, 4.68]; *p* = 0.01) (Figure 6A). Two studies [48, 49] investigated the effects of prolonged MT administration on changes in the GSH/GSSG ratio. Of these, one study [48] investigated the effects of prolonged MT administration on changes in the GSH/GSSG ratio, separately, in male and female groups. Using a random-effects model, we found that prolonged oral MT administration significantly enhanced the GSH/GSSG ratio (I^2^ = 53%; MD = 1.12; 95% CI [0.77, 1.47]; *p* < 0.00001) (Figure 6B).

### *Effects of MT on cathepsin B and cathepsin D*

**Figure 7:**
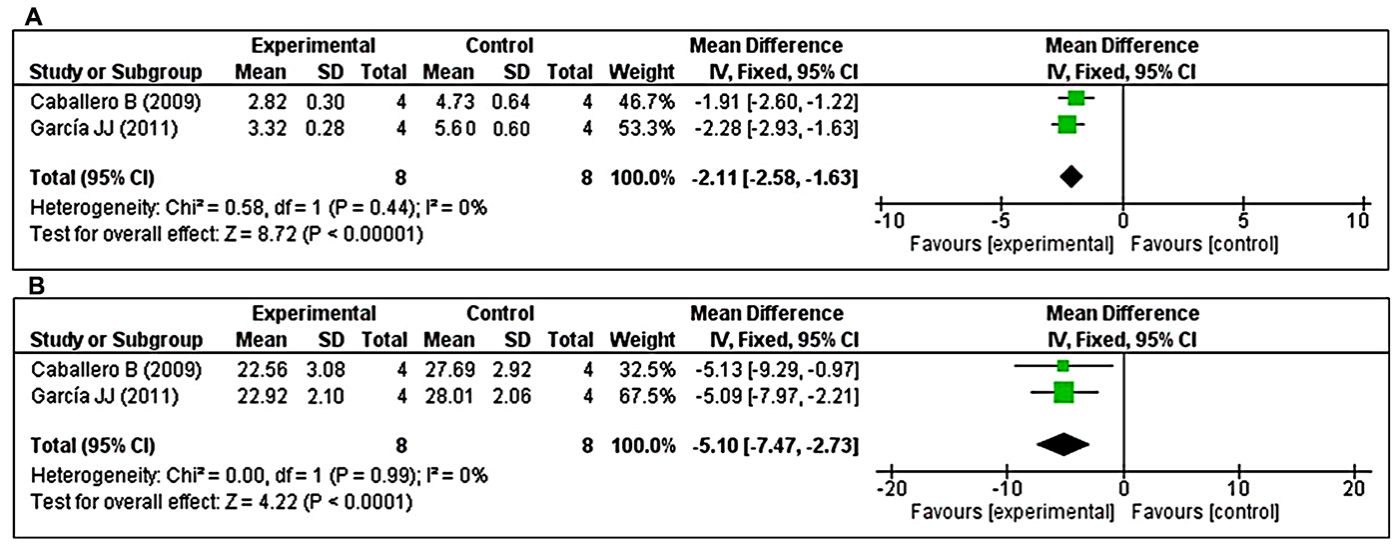
**Forest plot comparing changes in the levels of cathepsins B and D between MT-treated and vehicle-treated groups.** Compared with vehicle treatment, (**A**) cathepsin B and (**B**) cathepsin D levels were both significantly reduced in the MT-treated group. The unit for cathepsin B and cathepsin D are mU/mg, and U/mg, respectively. The prism represents the overall statistical results of the experimental data, squares represent the weight of each study, and horizontal lines represent the 95% CIs for each study. MT, melatonin; CI, confidence interval; SD, standard deviation; IV, independent variable.

The effects of MT on cathepsin B and cathepsin D were determined based on two studies each. With respect to cathepsin B, two studies [47, 49] investigated the effects of prolonged MT administration on changes in cathepsin B levels. Using a fixed-effects model, we found that prolonged oral MT administration significantly reduced the cathepsin B concentration (I^2^ = 0%; MD = –2.11; 95% CI [-2.58, –1.63]; *p* < 0.00001) (Figure 7A). Regarding cathepsin D, two studies [47, 49] investigated the effects of prolonged MT administration on changes in cathepsin D levels. Using a fixed-effects model, we found that prolonged oral MT administration significantly reduced the cathepsin D concentration (I^2^ = 0%; MD = –5.10; 95% CI [–7.47, –2.73]; *p* < 0.0001) (Figure 7B).

### Subgroup analysis

**Figure 8:**
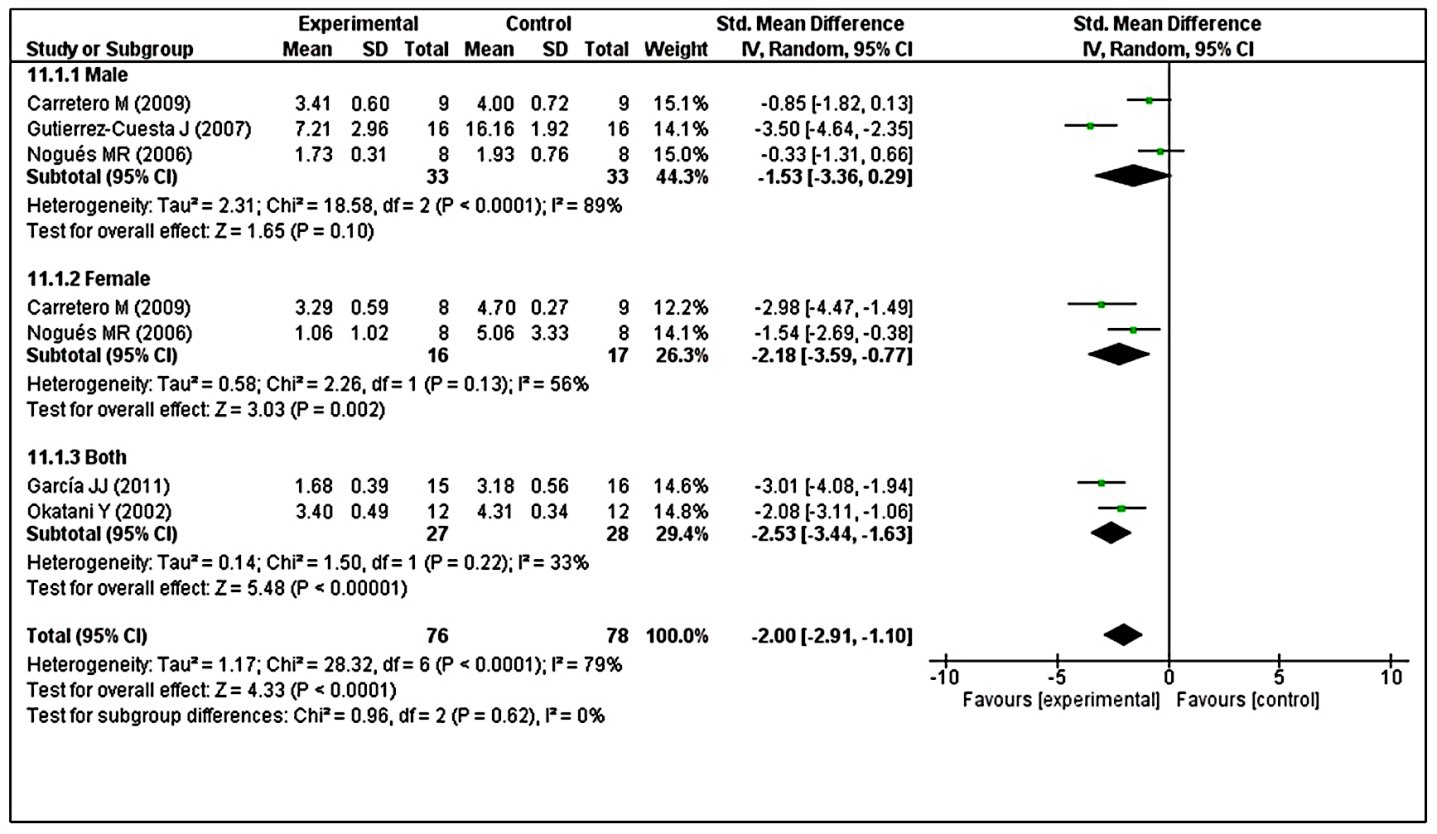
**Subgroup analysis of LPO levels stratified according to sex.** The test for subgroup differences showed no statistically significant subgroup effect (*p* = 0.62), indicating that sex did not modify the effect of MT intervention, compared with vehicle-treated groups. However, considerable heterogeneity was observed in the male (I^2^ = 89%) and female (I^2^ = 56%) subgroups. The unit for LPO almost in all studies is nmol/mg, except Nogues MR et al. (nmol/mL). The prism represents the overall statistical results of the experimental data, squares represent the weight of each study, and horizontal lines represent the 95% CIs for each study. LPO, lipid peroxidation; MT, melatonin; I, heterogeneity; CI, confidence interval; SD, standard deviation; IV, independent variable.

Subgroup analysis was performed to investigate heterogeneity among the studies. With regard to LPO, we found considerable heterogeneity (I^2^ = 79%). Thus, we performed post hoc subgroup analysis based on sex. Two studies [40, 49] used both male and female animals, but reported combined results. In contrast, three studies [41, 43, 48] reported results separately in male animals and two studies [41, 48] reported results separately in female animals. The test for subgroup differences indicated no statistically significant subgroup effect (*p* = 0.62), suggesting that sex did not modify the effect of MT compared with control intervention. Notably, the pooled effect estimates for male animals did not favor MT intervention (*p* = 0.10), although the pooled effect estimates for female animals and combined sex subgroups favored MT intervention (*p* = 0.002; *p* < 0.00001, respectively). However, there was substantial unexplained heterogeneity between trials involving male and female subgroups (males: I^2^ = 89%; females: I^2^ = 56%) (Figure 8). Furthermore, there was moderate heterogeneity in the combined sex subgroups (I^2^ = 33%), reduced heterogeneity in female animals (overall I^2^ = 79% to I^2^ = 56%), and enhanced heterogeneity in male animals (overall I^2^ = 79% to I^2^ = 89%). Therefore, the validity of the treatment effect estimate for male and female subgroups is uncertain because individual trial results were inconsistent.

**Figure 9:**
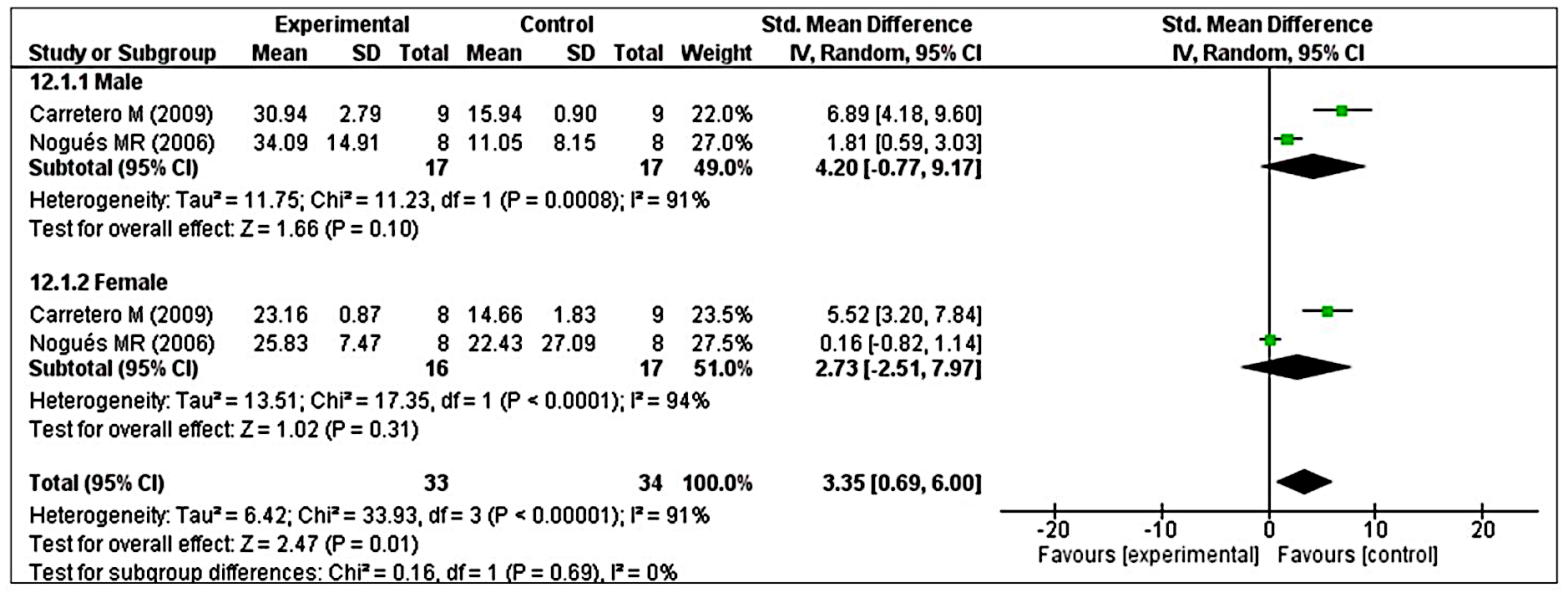
**Subgroup analysis of GRx levels stratified according to sex.** The test for subgroup differences showed a significant subgroup effect (*p* = 0.69), suggesting that sex did not modified the effect of MT intervention, compared with vehicle-treated groups. It is interesting to note that the pooled effect estimate for both subgroups favors MT intervention over the control intervention. The unit for GRx almost in all studies is nmol/mg, except Nogues MR et al. (nmol/mL). The prism represents the overall statistical results of the experimental data, squares represent the weight of each study, and horizontal lines represent the 95% CIs for each study. GRx, glutathione reductase; MT, melatonin; I, heterogeneity; CI, confidence interval; SD, standard deviation; IV, independent variable.

Analysis of GRx revealed considerable heterogeneity (I^2^ = 90%), so we performed post hoc subgroup analysis based on sex. Two studies reported male animals separately and two studies reported female animals separately [41, 48]. The test for subgroup differences indicated no statistically significant subgroup effect (*p* = 0.69), implying that sex could not significantly modified the effect of MT compared with control intervention. However, a smaller number of trials and animals contributed data to both male and female subgroups, meaning that the analysis may not be able to detect subgroup differences. It is interesting to note that the pooled effect estimate for both subgroups favors MT intervention over the control intervention (Figure 9).

### Sensitivity analysis

Sensitivity analysis by excluding individual studies revealed that the LPO (Supplementary Table 1), and GRx (Supplementary Table 4) results were not modified when compared to the overall effect and their heterogeneity, indicating that our results were not driven by any single study and that similar results could be obtained after excluding any of the included studies. The sensitivity analysis based on carbonylated protein indicated that heterogeneity decreased (I^2^ =0%, *p* = 0.81) when reference [43] was excluded; the adjusted MD was -3.00; 95% CI [-4.59, -1.40] (Supplementary Table 2). In accordance with GPx, the sensitivity analysis indicated that heterogeneity decreased (I^2^ =5%, *p* = 0.30) when reference [48] was excluded; the adjusted SMD was 2.61 95% CI [1.77, 3.46] (Supplementary Table 3). According to GSH/GSSH ratio, the sensitivity analysis indicated that heterogeneity decreased (I^2^ =27%, *p* = 0.24) when reference [48] was excluded; the adjusted MD was 1.25; 95% CI [0.93, 1.58] (Supplementary Table 5). Overall, sensitivity analysis demonstrated that the results produced in this meta-analysis were robust.

### Publication bias

Except for GRx activity (*p* = 0.0000, Egger's test) there was no evidence of publication bias for studies examining the effects of LPO (*p* = 0.0767, Egger's test; funnel plot: Supplementary Figure 1), and carbonylated protein (*p* = 0.7538, Egger's test). Using the trim-and-fill method, one potentially missing study was imputed for the analysis of GRx (Supplementary Figure 2). The imputed effect size of GRx was SMD 1.81; 95% CI [−1.12, 4.74].

### Systematic review of senescence-associated factors

### Effects of MT on senescence-associated molecular factors

NF-κB pathways are currently considered the primary causes of OS-induced CS [18]. Furthermore, OS induces nuclear translocation of NF-κB leading to its activation and the simultaneous generation of inflammatory factors such as tumor necrosis factor-α (TNF-α), interleukins, and chemokines [50–52]. Importantly, higher levels of NF-κB p50 subunits have been found in the nucleus in SAMP-8 mice at 10 months of age, indicating activation of this transcriptional factor in association with age-related enhanced OS [44]. In contrast, MT treatment reduced NF-κB p50 subunit levels in SAMP-8 mice and led to a clear reduction in nuclear localization [44]. Another study reported that pro-inflammatory mediators such as interleukin-1β, TNF-α, and interleukin-6 expression were elevated in SAMP-8 at 10 months of age, particularly in the hippocampal region [53]. Consistent with these findings, Rodríguez et al. also reported that pro-inflammatory cytokines, mainly interferon gamma and TNF-α, were elevated in plasma from SAMP8 mice at 10 months of age. These elevated levels were counteracted by prolonged MT treatment [42]. Similarly, Gutierrez-Cuesta et al. demonstrated that prolonged MT treatment reduced NF-κB in the 10-month-old SAMP8 mouse brain, although to a lesser extent [46]. Thus, reduced longevity could be due to sustained activation of NF-κB, which leads to age-related diseases [54].

Cdk5 may be an important link between OS and CS [19]. In SAMP8 mice, the activation of Cdk5 was significantly increased at 10 months of age [43], the age of senescence onset [55]. Importantly, oral MT administration (10 mg/kg, from 2 to 10 months of age) reduced this activation [43], suggesting that a reduction in CS leads to diminished age-related neurodegeneration. In the same study, MT lowered the levels of Cdk5/p35 and active glycogen synthase kinase 3β (GSK3β), and enhanced the levels of inactive GSK3β (Ser9), in 10-month-old SAMP8, thus causing reduced tau hyperphosphorylation [43]. These findings indicate that Cdk5 and GSK3β might be interrelated and may contribute to senescence.

p53 is a pro-apoptotic transcriptional factor that is induced by OS and is considered important in the induction of CS [20]. Surprisingly, the deacetylation of p53 through sirtuin-1 (SIRT1) may suppress CS and apoptosis induced by OS [56]. In previous time-course studies, SIRT1 expression showed progressive reduction from 3 to 12 months of age in SAMP8 mice [57]. Likewise, compared with untreated animals, oral administration of MT treatment (10 mg/kg) in drinking water induced significant enhancement of SIRT1 expression and reduced the expression of acetylated p53 in SAMP8 mice [46]. Western blot analysis also showed that a gradual age-dependent enhancement of p53 levels was reduced by chronic MT treatment in the SAMP8 mouse brain [47]. In addition, p53-mediated suppression of apoptosis inhibitory proteins, such as Bcl-2, was augmented by long-term MT treatment in SAMP8 mice [47].

### *Effects of MT on senescence-associated morphological factors*

Data from several studies suggest that senescence accelerates gliosis during aging, which might contribute to neuronal dysfunction [58]. Nissl staining analysis showed that gliosis in cortical layers was reduced following 9 months of dietary intake of MT, compared with vehicle-treated SAMP8, suggesting a neuroprotective effect of MT during aging [43]. Moreover, cresyl violet staining in the hippocampal CA1 and CA3 regions of SAMP8 showed that subcutaneous MT treatment (1 mg/kg/day) increased the density of pyramidal neurons, when compared with untreated mice (*P* < 0.01) [45]. No differences were observed between the early-age MT treatment group (SAMP8 age = 4–8 months) and the late-age MT treatment group (SAMP8 age = 8–11 months). Furthermore, in hippocampal CA3 regions, the early-age MT treatment group showed markedly greater numbers of pyramidal neurons, compared with the late-age MT treatment group (*P* < 0.05). No differences were observed between the late-age MT treatment and control groups [45].

## DISCUSSION

This study systematically reviewed previous research concerning the protective effect of MT against brain aging in the SAMP8 mouse model, which shares similar pathophysiological features with humans in terms of brain aging [22]. The principal aim was to provide evidence for the therapeutic potential of MT to promote healthy aging and prevent age-related diseases by maintenance of physiological homeostasis between OS and CS. In recent years, several feasible interventions (e.g., MT) to delay aging have been studied in SAMP8 mice, although these have led to some conflicting results [40, 45, 47]. Furthermore, no single systematic review and meta-analysis has been conducted to evaluate the efficacy of MT in this context. To our knowledge, this is the first systematic review and meta-analysis concerning the effects of MT on OS-induced brain aging in SAMP8 mice. Our results suggest that prolonged MT treatment increases oxidative stability and antioxidative enzyme activity, reduces lysosomal enzymatic function, and regulates senescence-associated factors in the brain. Among the indicators and oxidant/antioxidant factors considered during investigation of this topic, LPO, carbonylated protein, CAT, GPx, SOD, GRx, GSH/GSSG ratio, cathepsin B, and cathepsin D have been evaluated in meta-analyses of animal trials. Additionally, senescence-associated molecular and morphological factors (e.g., NF-κB, Cdk5, and p53) were systematically reviewed in the SAMP8 mouse model.

Despite extensive efforts, we were only able to include 10 studies in this review. Of these, five aimed to evaluate the effects of prolonged MT treatment on changes in LPO levels. The meta-analysis results showed that MT treatment led to a significant reduction in the amount of LPO (*p* < 0.0001), indicating that MT was effective against oxidative degradation of lipids during aging. These results are consistent with the findings of Verma et al*.* (2020), who found that MT supplementation could alleviate age-related enhancement of LPO through the maintenance of normal redox homeostasis [59]. Taken together, these data indicate that the age-dependent decline in pineal MT synthesis may contribute to increased production of LPO end products, and that MT supplementation can reverse these detrimental effects. Mitochondrial LPO exhibited a sharp enhancement at 10 months of age in SAMP8 mice [49]. Thus, we assessed the effects of MT at ≥ 10 months of age. However, these data must be interpreted cautiously because there was substantial unexplained heterogeneity between trials within male and female subgroups (male: I^2^ = 89%; female: I^2^ = 56%). Therefore, the validity of the treatment effect estimate for male and female animals is uncertain because individual trial results were inconsistent.

A prior study by Carney and colleagues [60] revealed age-related increases in carbonyl concentrations in an animal model of brain aging. The formation of carbonyl protein by free radicals (widely regarded as an indicator of oxidative damage) is primarily involved in cell damage during normal aging and aging-related diseases [15, 60]. In the brain, oxidized protein exhibits complex connections with the levels of antioxidative enzymes, proteolytic elimination of oxidized proteins, and generation of pro-oxidant substances [61]. Consistent with published literature, our meta-analysis revealed that prolonged MT treatment significantly reduced the formation of carbonylated proteins, compared with untreated SAMP8 mice (*p* = 0.03), suggesting that age-related enhancement of oxidative brain damage could be prevented by reducing carbonylated protein levels. Notably, the positive results might have been due to a high degree of heterogeneity (I^2^ = 93%) among the included studies. Hence, the validity of the treatment effect estimate for this outcome is dubious because individual study results were inconsistent.

Accumulation of high protein content was observed in the aged SAMP8 mouse brain [44], presumably due to neurodegeneration and age-related OS [43]. These aggregations may result from disturbances in typical enzymatic activity caused by OS during brain aging. Changes in the activities of antioxidant enzymes, including CAT, led to elevated levels of OS in SAMP8 mice until 10 months of age; this may be one cause of senescence-related impairments and degeneration in the brain [43]. Surprisingly, no differences were found in CAT activity between the MT and vehicle treatment groups (*p* = 0.32). This outcome is contrary to that of Tütüncüler et al., who found that the administration of exogenous MT effectively protected against brain injury by increasing CAT activity [62]. Conversely, Gutierrez-Cuesta et al. reported that long-term MT treatment significantly reduced CAT expression in SAMP8 mice, compared with untreated SAMP8 mice [43]. One possible explanation is that long-term MT treatment differentially interacted with CAT activity. Specifically, prolonged presence of MT led to low OS, suggesting a linear relationship between OS and CAT activity [43]. Additionally, we demonstrated that MT supplementation significantly enhanced GPx activity (*p* < 0.00001) in SAMP8 mice. This result implies that MT administration promoted antioxidant enzyme activity in the brain and may thereby provide indirect protection against free radical injury [63]. Surprisingly, MT treatment did not affect SOD activity in the SAMP8 brain (*p* = 0.07), although randomized controlled trials of MT showed marked increases in SOD expression [64]. This result contributes to the ongoing controversy regarding the link between SOD activity and aging [65], whereby SOD activity was not influenced by MT administration [62]. In contrast, GRx activity in SAMP8 mice was significantly reduced during aging, and MT treatment significantly enhanced GRx activity compared with the untreated group in the same mouse model (*p* = 0.01). These findings indicated that the decline in GRx activity may play a significant role in brain damage. Notably, the results may be biased due to insufficient trials (two studies per subgroup) and numbers of animals in each subgroup (male = 34; female = 33), so the covariate distribution is problematic for this subgroup analysis. Furthermore, the GSH/GSSG ratio significantly decreases with age [66], although our meta-analysis results showed that long-term MT treatment significantly enhanced the GSH/GSSG ratio (*p* < 0.00001) in SAMP8 mice. This result is in agreement with the findings of Alzoubi et al., who demonstrated that MT reduced OS by enhancing the GSH/GSSG ratio in the rat hippocampus [67].

Cathepsins B and D are the main lysosomal proteases and are abundantly expressed in the brain [68, 69]. It is unsurprising that the levels of these proteases increase with age, as well as in several age-dependent neurodegenerative diseases [70, 71]. The results from previous studies revealed that the levels of these proteases were increased in the aged SAMP8 mouse brain [49]. In particular, cathepsin D is regarded as a marker of aging [72]. Our meta-analysis results showed that MT significantly reduced both cathepsin B (*p* < 0.00001) and cathepsin D (*p* < 0.0001) levels in the brains of SAMP8 mice, indicating that lysosomal impairment may be involved in brain aging. This finding broadly supports the work of other studies in this area that link lysosomal impairment with aging [73, 74].

Most recently, Bernal et al. showed that the activation of NF-κB led to CS acceleration via telomere dysfunction [75]. In addition, an age-related increase in the inflammatory response is considered a hallmark of CS [76]. Moreover, lysosomal enzymes (e.g., cathepsin B) indirectly activate NF-κB, resulting in microglial senescence-induced brain aging [77]. In our systematic review, we found that prolonged MT treatment reduced NF-κB p50 levels in SAMP8 mice, and markedly reduced nuclear localization indicating lower NF-κB activity [44]. On the basis of these data, we infer that potent NF-κB activation and its associated inflammatory response may contribute to aging processes linked to accelerated senescence in SAMP8 mice. In contrast, prolonged MT treatment may suppress CS and age-related disorders by downregulation of NF-κB signaling. These results are in agreement with the findings of Fang et al*.*, who showed that MT prevented senescence through inhibition of NF-κB signaling pathways [78].

Previously, Lee et al*.* demonstrated that Cdk5 stabilizes and activates p53, a common pathway that promotes CS [79], thereby supporting the notion that Cdk5 is involved in senescence induction [19, 80]. The activation of Cdk5 and the levels of active GSK3β (Tyr216) were significantly increased at 10 months of age in SAMP8 mice, although MT mitigated these changes [43], thus implying that Cdk5 and GSK3β might be related and may contribute to senescence. These results reflect the findings of Liu et al., who reported the inhibition of Cdk5 and GSK-3β activities, as well as reduction of Alzheimer's disease-like pathology, in senescence-accelerated mice [81].

OS plays a critical role in the activation of the p53 transcriptional responses, which is responsible for the regulation of both CS and aging [20]. Surprisingly, the deacetylation of p53 through SIRT1 may suppress both OS-induced CS and OS-induced apoptosis [56]. The expression of Bcl-2 is enhanced in human fibroblasts naturally during senescence and upon induction of OS-induced senescence-like growth arrest, implying its role in maintenance of their extended viability [82, 83]. These findings suggested that elevated OS during the aging process dysregulates apoptosis, thereby inducing CS. They also corroborate previous results, in that senescent cells do not readily undergo apoptosis [84]. Our data suggest that oral MT treatment (10 mg/kg) induced significant elevation of SIRT1 expression and reduced the expression of acetylated p53 in SAMP8 mice [46]. In accordance with the present results, earlier findings demonstrated that SIRT1 overexpression led to reduction of p53 gene expression [85, 86]. Additionally, Liu et al. demonstrated that SIRT1 reversed senescence via attenuation of OS-induced apoptosis and promotion of p53 degradation [86].

## CONCLUSIONS AND FUTURE DIRECTIONS

This study was performed to evaluate the ability of MT to foster healthy aging and counteract age-related disorders by inhibition of pathways involved in accelerated senescence. The findings of this study suggest that orally administered long-term MT treatment reduces formation of LPO, carbonylated proteins, and lysosomal proteases. Moreover, it increases the activities of GPx and GRx, as well as the GSH/GSSG ratio. Although the potential source of heterogeneity has been investigated through leave-one-out sensitivity analyses, our findings have some limitations with respect to generalizability. For instance, there was substantial unexplained heterogeneity between trials within male and female subgroups. If the debate is to be moved forward, a better understanding of sex-specific long-term MT effects is needed. Another important result was that the CAT and SOD activities remain unchanged with MT intervention. Further research should explore how short-term and long-term MT interventions differentially impact CAT and SOD activities during aging. The second major finding was that MT administration can regulate senescence-associated molecular and morphological factors. Although we only narratively described these results because of the small number of studies, the findings offer some insight into how MT non-destructively regulates CS. Further studies would be useful regarding validation of the anti-senescence role of MT. Finally, our quality assessment using the SYRCLE RoB tool indicated that all included studies had considerable methodological limitations, a high risk of selection bias, and unclear risk of detection bias. Information regarding key measures essential for bias reduction (e.g., allocation concealment, random outcome assessment, and blinding) was often missing or insufficiently reported. Unfortunately, this is common in animal studies and limits our ability to draw plausible conclusions [87, 88]. We strongly recommend improvements to the reporting system for animal models to reduce the RoB; recently developed guidelines should be followed to enhance the quality of animal studies [89, 90].

## MATERIALS AND METHODS

### Search strategy

Research articles reporting the effects of MT on the brain in SAMP8 were included in this systematic review and meta-analysis. The literature search was executed using keywords such as ‘melatonin’ in combination with ‘brain, aging, CS, and SAMP8’ in the following databases: PubMed, Embase, and CINAHL for studies published until August 2019. The reference lists of the included studies and those of relevant reviews were examined to identify additional relevant studies. The in-depth search strategy performed in the PubMed electronic database is shown in Supplementary Table 6. No limits on language or publication date were used.

### Inclusion and exclusion criteria

A systematic review and meta-analysis of research assessing the effects of exogenous MT on OS in the brain or plasma of SAMP8 mice was performed if the parameter of interest was reported in two or more studies. The systematic review focused on senescence-associated molecular and morphological factors. The meta-analysis focused on the levels/activities of brain/plasma OS markers (e.g., LPO, carbonylated protein, SOD, GPx, GRx, CAT, and GSH/GSSG ratio), as well as the levels/activities of lysosomal enzymes (e.g., cathepsins B and D), which were compared between vehicle control and MT treatment groups. Studies were excluded if they did not use SAMP8 mice, used interventions other than MT, were *ex vivo* or *in vitro* experiments, were unrelated outcome or review format, or were not published in English (Supplementary Table 7).

### Study selection

After removal of duplicates, all unique trials were imported into a Rayyan-a web application [91] to allocate the references randomly. Next, two authors individually screened the titles and abstracts to select relevant studies from the randomly allocated references. Finally, the full texts of the selected articles were evaluated to identify trials that fulfilled our eligibility criteria. Any disagreement concerning study selection was settled by consultation with a third author. Notably, screening for the presence or absence of specific outcome measures was not performed during this phase because some outcome measures were not described in the abstract.

### Data extraction

Two authors individually extracted the data from each of the included studies. Information related to the authors, publication year, age, sex, sample size, intervention (i.e., dose, route of administration, and duration), and outcome measures were extracted. For studies with multiple interventions, only data from the control and MT treatment groups were considered in this analysis. If published outcome data were incomplete, attempts were made to contact the study authors to obtain the original data. A reminder was sent by email to authors who had not responded within 2 weeks. If efforts to acquire the original data failed, the article was eliminated from the meta-analysis. If the data were only presented graphically, GetData Graph Digitizer (http://getdata-graph-digitizer.com/) was used to extract numerical data from graphs or figures.

### Assessment of methodological quality

The RoB in the included articles was evaluated by two independent reviewers using the SYRCLE RoB tool [92], which was developed based on the Cochrane RoB tool [93] to evaluate aspects of bias specifically encountered in animal intervention studies. The tool contains 10 items related to six types of bias (selection, performance, detection, attrition, reporting, and other bias). Responses of ‘yes’, ‘no’, and ‘unsure’ indicated low, high, and unclear RoB, respectively.

### Data analysis

The experimental and control group data from the included studies were extracted and entered into the Review Manager software (RevMan 5.3, The Nordic Cochrane Centre, Copenhagen, Denmark). A meta-analysis was performed when at least two studies were analogous in terms of population, intervention, comparison, outcome process, and study design, and when these studies provided relevant data. For effect size analysis, the MD was used when the outcome measures of all studies employed the same scale, and the SMD was used when the studies assessed the same outcome by means of distinct measurements. For both strategies, 95% CIs were calculated. Large SMD effect size was considered 0.8, moderate 0.5, and small 0.2 [94]. The I^2^ test was used to assess heterogeneity among studies. A fixed-effects model was used for the meta-analysis when I^2^ was ≤ 50%, and a random-effects model was used when I^2^ was > 50%, indicating substantial heterogeneity [95]. Subgroup analyses were performed only when subgroups contained at least two independent comparisons. Whenever three or more studies were included, a leave-one-out sensitivity analysis was performed by iteratively removing 1 study at a time to confirm that our findings were not driven by any single study, as well as, to assess potential sources of heterogeneity [96]. Publication bias was investigated via Egger's test (≥ 4 studies) and visual inspection of funnel plots (≥ 5 studies) using Stata/SE software, Version 16.0 (Stata Corp., College Station, TX) (*p* < 0.05) [97, 98]. Whenever publication bias was detected either funnel plot asymmetry or Egger’s regression test, the trim and fill method was used to calculate the effect size by estimating the number of missing studies [99].

## Supplementary Material

Supplementary Figures

Supplementary Tables
