# Supplementary material for: Differential role of melatonin in healthy brain aging: a systematic review and meta-analysis of the SAMP8 model
Source: Aging (Albany NY). 2021 Apr 2;13(7):9373–97. doi: 10.18632/aging.202894 (PMC8064193; doi:10.18632/aging.202894)
Supplement: Supplementary Figures [file aging-13-202894-s001.pdf]

SUPPLEMENTARY FIGURES

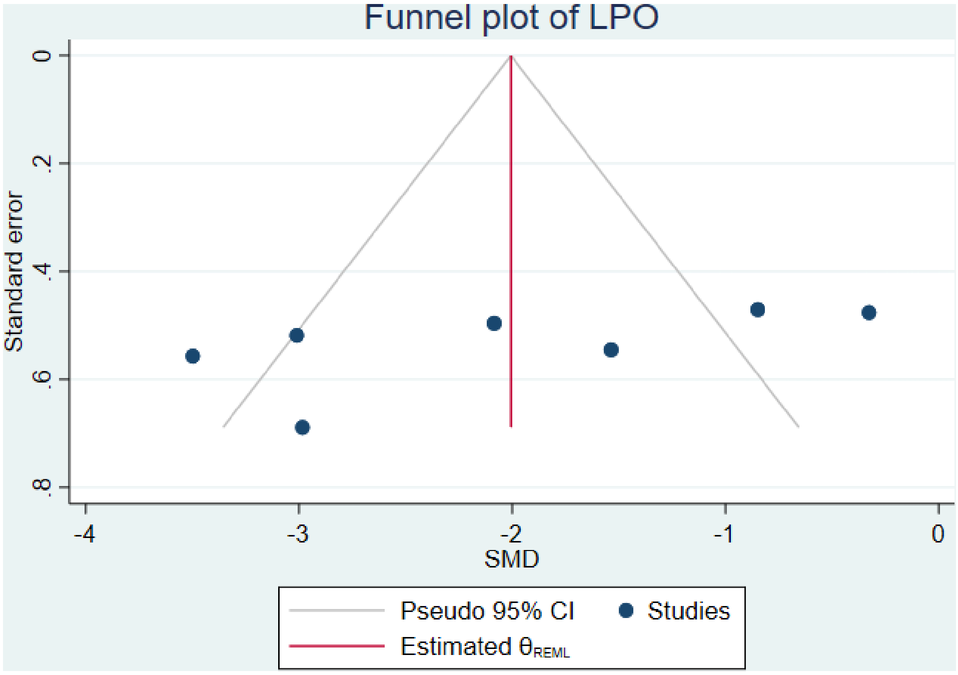

Supplementary Figure 1. Symmetric funnel plot in studies reporting the impact of MT on LPO levels. SMD, standardized mean difference; MT, melatonin.

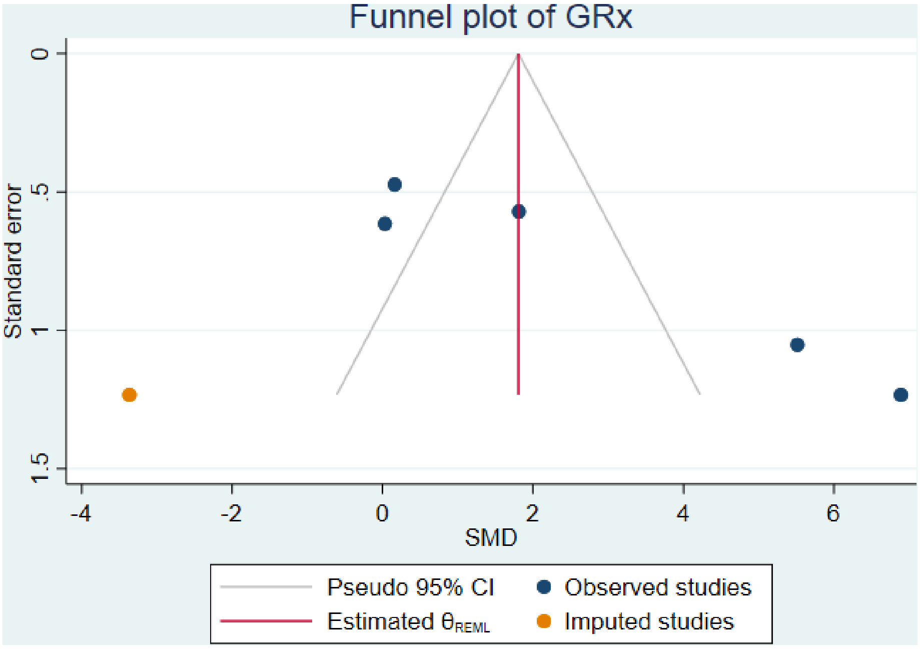

Supplementary Figure 2. Asymmetric funnel plot showing the publication bias in studies reporting the impact of MT on GRx levels. SMD, standardized mean difference; MT, melatonin; GRx, glutathione reductase.
