# Supplementary material for: Differential role of melatonin in healthy brain aging: a systematic review and meta-analysis of the SAMP8 model
Source: Aging (Albany NY). 2021 Apr 2;13(7):9373–97. doi: 10.18632/aging.202894 (PMC8064193; doi:10.18632/aging.202894)
Supplement: Supplementary Tables [file aging-13-202894-s002.pdf]

## SUPPLEMENTARY TABLES

**Supplementary Table 1. Leave-one-out sensitivity analyses on LPO.**

| | Pooled SMD [95% CI] | Between-study heterogeneity |
|---|---|---|
| **Overall** | -2.00[-2.91, -1.10] | $p$ < 0.0001; $I^2$=79% |
| **Omitted study** | | |
| Carretero M (2009) female | -1.86[-2.85, -0.88] | $p$ < 0.0001; $I^2$=81% |
| Carretero M (2009) male | -2.21[-3.20, -1.22] | $p$ = 0.0003; $I^2$=78% |
| Garcia J (2011) | -1.99[-3.08, -0.91] | $p$ < 0.0001; $I^2$=82% |
| Gutierrez-Cuesta J (2007) | -1.75[-2.63, -0.86] | $p$ = 0.002; $I^2$=74% |
| Nogues MR (2006) female | -2.08[-3.14, -1.03] | $p$ < 0.0001; $I^2$=82% |
| Nogues MR (2006) male | -2.29[-3.13, -1.44] | $p$ = 0.005; $I^2$=70% |
| Okantani Y (2002) | -1.83[-2.81, -0.85] | $p$ = 0.0003; $I^2$=78% |

**Supplementary Table 2. Leave-one-out sensitivity analyses on carbonylated protein.**

| | Pooled MD [95% CI] | Between-study heterogeneity |
|---|---|---|
| **Overall** | -5.74[-11.03, -0.44] | $p$ < 0.00001; $I^2$=93% |
| **Omitted study** | | |
| Caballero B (2008) | -6.38[-13.25, 0.48] | $p$ < 0.00001; $I^2$=95% |
| Garcia JJ (2011) | -6.40[-13.29, 0.49] | $p$ < 0.00001; $I^2$=95% |
| Gutierrez-Cuesta J (2007) | -3.00[-4.59, -1.40] | $p$ = 0.81; $I^2$=0% |
| Okantani Y (2002) | -6.84[-13.44, -0.24] | $p$ < 0.00001; $I^2$=92% |

**Supplementary Table 3. Leave-one-out sensitivity analyses on GPx.**

| | Pooled SMD [95% CI] | Between-study heterogeneity |
|---|---|---|
| **Overall** | 3.33[1.89, 4.78] | $p$ = 0.06; $I^2$=65% |
| **Omitted study** | | |
| Carretero M (2009) female | 3.53[0.91, 6.14] | $p$ = 0.02; $I^2$=81% |
| Carretero M (2009) male | 2.61[1.77, 3.46] | $p$ = 0.30; $I^2$=5% |
| Okantani Y (2002) | 4.04[2.38, 5.71] | $p$ = 0.20; $I^2$=40% |

**Supplementary Table 4. Leave-one-out sensitivity analyses on GRx.**

| | Pooled SMD [95% CI] | Between-study heterogeneity |
|---|---|---|
| **Overall** | 2.59[0.50, 4.68] | $p$ < 0.00001; $I^2$=90% |
| **Omitted study** | | |
| Caballero B (2009) | 3.35[0.69, 6.00] | $p$ < 0.00001; $I^2$=91% |
| Carretero M (2009) female | 1.87[-0.13, 3.87] | $p$ < 0.0001, $I^2$=88% |
| Carretero M (2009) male | 1.65[-0.16, 3.45] | $p$ = 0.0001, $I^2$=86% |
| Nogues MR (2006) female | 3.35[0.62, 6.09] | $p$ < 0.00001, $I^2$=90% |
| Nogues MR (2006) male | 2.92[0.01, 5.83] | $p$ < 0.00001, $I^2$=92% |

**Supplementary Table 5. Leave-one-out sensitivity analyses on GSH/GSSH ratio.**

| | Pooled MD [95% CI] | Between-study heterogeneity |
|---|---|---|
| **Overall** | 1.12[0.77, 1.47] | $p = 0.12$; $I^2$=53% |
| **Omitted study** | | |
| Carretero M (2009) female | 0.99[0.66, 1.33] | $p = 0.21$; $I^2$=36% |
| Carretero M (2009) male | 1.25[0.93, 1.58] | $p = 0.24$; $I^2$=27% |
| Garcia JJ (2011) | 1.12[0.44, 1.81] | $p = 0.04$; $I^2$=76% |

**Supplementary Table 6. PubMed search strategy (01 August 2019).**

| Search | Query | Items found |
|---|---|---|
| #1 | ("melatonin"[MeSH Terms] OR "melatonin"[All Fields] OR "n acetyl 5 methoxytryptamine"[All Fields]) | 25030 |
| #2 | (((("brain"[MeSH Terms] OR "brain"[All Fields]) AND ("aging"[MeSH Terms] OR "aging"[All Fields] OR "agings"[All Fields] OR "ageing"[All Fields] OR "ageings"[All Fields])) OR ("sensation"[MeSH Terms] OR "sensation"[All Fields] OR "senses"[All Fields]) OR ("geriatrics"[MeSH Terms] OR "geriatrics"[All Fields] OR "geriatric"[All Fields] OR "gerontol"[All Fields])) AND "cellular senescence"[MeSH Terms]) OR "cellular senescence"[All Fields] | 18,940 |
| #3 | (("rodentia"[MeSH Terms] OR "rodentia"[All Fields] OR "rodent"[All Fields] OR ("rats"[MeSH Terms] OR "rats"[All Fields] OR "rat"[All Fields]) OR ("mice"[MeSH Terms] OR "mice"[All Fields]) OR ("mice"[MeSH Terms] OR "mice"[All Fields] OR "mouse"[All Fields]) OR ("rats"[MeSH Terms] OR "rats"[All Fields] OR "rattus"[All Fields]) OR "mus"[All Fields]) AND "SAM"[All Fields]) OR "samp"[All Fields] OR "SAMP8"[All Fields] OR "SAMP10"[All Fields] OR (("aging"[MeSH Terms] OR "aging"[All Fields] OR "senescence"[All Fields]) AND ("mice"[MeSH Terms] OR "mice"[All Fields])) | 53,179 |
| #4 | #2 OR #3 | 67,390 |
| #5 | #1 AND #4 | 303 |

**Supplementary Table 7. Explanations for the full-text article exclusions.**

| SL/NO | Title | Reasons |
|---|---|---|
| 1 | Gutierrez-Cuesta J, Tajes M, Jimenez A, Camins A, Pallas M. [Effects of melatonin in the brain of the senescence-accelerated mice-prone 8 (SAMP8) model]. Rev Neurol. 2011; 52: 618–22. | Review |
| 2 | Asai M, Ikeda M, Akiyama M, Oshima I, Shibata S. Administration of melatonin in drinking water promotes the phase advance of light-dark cycle in senescence-accelerated mice, SAMR1 but not SAMP8. Brain Res. 2000; 876: 220–4. | Unrelated outcome |
| 3 | Lardone PJ, Alvarez-García Ó, Carrillo-Vico A, Vega-Naredo I, Caballero B, Guerrero JM, Coto-Montes A. Inverse correlation between endogenous melatonin levels and oxidative damage in some tissues of SAM P8 mice. Journal of Pineal Research. 2006; 40: 153–7. | Wrong study organ |
| 4 | Rosenfeld SV, Togo EF, Mikheev VS, Popovich IG, Khavinson VK, Anisimov VN. Effect of epithalon on the incidence of chromosome aberrations in senescence-accelerated mice. Bull Exp Biol Med. 2002; 133: 274–6. | Wrong study design |
| 5 | Shibata S, Asai M, Oshima I, Ikeda M, Yoshioka T. Melatonin normalizes the re-entrainment of senescence accelerated mice (SAM) to a new light-dark cycle. Adv Exp Med Biol. 1999; 460: 261–70. | Unavailable |
| 6 | Parisotto EB, Vidal V, García-Cerro S, Lantigua S, Wilhelm Filho D, Sanchez-Barceló EJ, Martínez-Cué C, Rueda N. Chronic Melatonin Administration Reduced Oxidative Damage and Cellular Senescence in the Hippocampus of a Mouse Model of Down Syndrome. Neurochem Res. 2016; 41: 2904–13. | Wrong animal model |
| 7 | Morioka N, Okatani Y, Wakatsuki A. Melatonin protects against age-related DNA damage in the brains of female senescence-accelerated mice. J Pineal Res. 1999; 27: 202–9. | Wrong animal model |
| 8 | Cristòfol R, Porquet D, Corpas R, Coto-Montes A, Serret J, Camins A, Pallàs M, Sanfeliu C. Neurons from senescence-accelerated SAMP8 mice are protected against frailty by the sirtuin 1 promoting agents melatonin and resveratrol. J Pineal Res. 2012; 52: 271–81. | *Ex-vivo* |
